# Xanthomes tendineux et tubéreux révélant une hypercholestérolémie familiale

**DOI:** 10.11604/pamj.2013.15.49.2636

**Published:** 2013-06-09

**Authors:** Wafae Raffas, Badreddine Hassam

**Affiliations:** 1 Service de Dermatologie, CHU Ibn Sina, Université Med V, Souissi, Rabat, Maroc

**Keywords:** Xanthomes tubéreux, nodules sous-cutanés, pseudotumeurs, lipoprotéines, troubles lipidiques, hypercholestérolémie familiale, tuberous xanthomas, subcutaneous nodules, pseudotumor, lipoproteins, lipid disorders, familial hypercholesterolemia

## Image en medicine

Les xanthomes sont des pseudotumeurs bénignes, le plus souvent liées à un trouble du métabolisme des lipoprotéines et qui en imposent le dépistage. Plusieurs types anatomocliniques sont décrits, s'associant chacun à des troubles lipidiques précis de sévérité variable. La précocité du diagnostic conditionne le pronostic et permet de prévenir, dépister et traiter les complications cardiovasculaires prématurées. En l'absence de dyslipidémie, un syndrome lymphoprolifératif ou une gammapathie monoclonale doivent être recherchés. Nous rapportons l'observation d'un patient de 29 ans, qui consultait pour des nodules indolores multiples des membres supérieurs évoluant depuis 5 ans. On notait une hypercholestérolémie chez la mère sans notion de lésions cutanées ni d'accident vasculaire prématuré. L'examen objectivait des xanthomes tubéreux en regard des coudes, et des xanthomes tendineux en regard des articulations métacarpo-phalangiennes de la main droite. On ne trouvait pas d'arc cornéen, ni de xanthélasma. Le reste de l'examen somatique était sans anomalies. L'examen histologique confirmait le diagnostic de xanthome en montrant un infiltrat dermique riche en histiocytes spumeux. Un bilan lipidique objectivait des taux de cholestérol total à 5,5g/l, de cholestérol LDL à 4,9g/l, et des triglycérides normaux. Le bilan thyroïdien, la radiographie thoracique, l’électrocardiogramme, l’échographie cardiaque et l’écho-Doppler des vaisseaux du cou et des membres étaient sans anomalies. Le diagnostic d'hypercholestérolémie familiale de type IIa selon la classification de Fredrickson était retenu. Le patient était pris en charge par des mesures hygiéno-diététiques associées à un traitement par simvastatine. Une chirurgie d'exérèse était réalisée pour le xanthome volumineux du coude droit.

**Figure 1:**
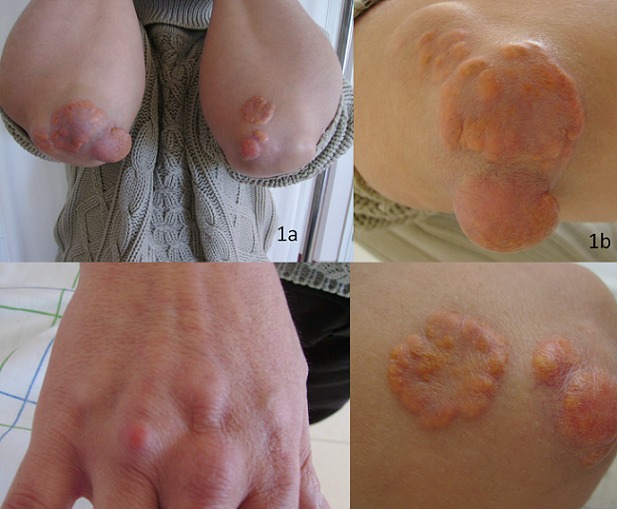
Xanthomes tubéreux. Nodules jaunâtres lisses coalescents par endroits réalisant un aspect annulaire au niveau des coudes (vue d'ensemble 1b. 1c. details)

